# Research highlight for issue 8: disease evolution and ecology across space

**DOI:** 10.1111/eva.12201

**Published:** 2014-09-29

**Authors:** Britt Koskella

How infectious disease spreads both from individual to individual and across a landscape will depend upon many inter-related factors, including the genetic composition of host and pathogen populations, the pathogen transmission rate, host density, population connectivity, and the evolutionary response of both host and pathogen over time. As such, the study of infectious disease straddles a number of fields and approaches. A key advance in the field has come from incorporation of spatial structure into both theoretical and empirical studies of the epidemiology, evolution, and ecology of disease. Researchers taking this approach were recently brought together for a workshop entitled ‘Spatial evolutionary epidemiology’ in Montpellier, France organized by Sébastien Lion and Sylvain Gandon from the Centre d’Écologie Fonctionnelle et Évolutive (CEFE). The workshop spanned topics from infection genetics in Daphnia, to cooperation and conflict in microbial populations, to the utility of spatial epidemiological models for designing better crop planting strategies, and emphasized the broader need to think about the importance of spatial heterogeneity in order to predict the spread and evolution of pathogens.

The recent literature includes a number of studies at the cutting edge of this field. For example, a model based on interactions between bacteria and their bacteriophage parasites by Ben Ashby and collaborators has demonstrated the importance of spatial structure in shaping the breadth of host resistance. They find that hosts and parasites from spatially structured populations should be less constrained by costs associated with ‘generalism’ than those from well-mixed populations, and therefore, that spatial structure is likely to increases the breadth of host resistance/parasite infectivity, especially when this increased breadth carries a significant fitness cost (Ashby et al. 2014). Another potential effect of spatial structure on host-parasite interactions is through changing rates of infection, as opposed to infection range. A recent empirical study of bacteria and phages by Pavitra Roychoudhury and coauthors experimentally evolved phages on spatially structured agar plates for 550 phage generations and found increased phage fitness was associated with a mutation conferring slower phage adsorption rate to bacterial host cells. This is in line with theoretical work suggesting spatial structure may lead to reduced parasite infectivity (e.g., Boots and Sasaki 1999) and builds upon previous empirical work from nonmicrobial systems in support of this theory. Furthermore, the authors develop a system-specific, spatially explicit model to explain how low attachment probability might lead to increased phage fitness through higher plaque density as a result of trade-offs between phage diffusion and adsorption (Roychoudhury et al. 2014).

Studying spatial structure in natural populations is of course a more challenging goal, as one loses the ability to control for the type of heterogeneity across which a study is performed. Despite these potential limitations, recent work by Jussi Jousimo, Ayco Tack, and collaborators examined the impact of connectivity among over 4000 populations of the plant, *Plantago lanceolata*, on resistance to a co-occurring fungal pathogen, *Podosphaera plantaginis*, over a 12-year period. Using this large spatial dataset, the authors were able to explain the unexpected observation that highly connected populations typically showed lower pathogen prevalence and higher pathogen extinction than more isolated populations. They demonstrate that hosts from highly connected populations are in fact more resistant to the pathogen than those from more isolated populations, most likely as a result of stronger parasite-mediated selection over time (Jousimo et al. 2014). This work highlights the importance of incorporating host and pathogen evolution into models predicting the spread of disease across space, and emphasizes the potential for differential parasite-mediated selection across a host metapopulation.

Finally, work by David Rasmussen and colleagues has demonstrated the strength of incorporating spatial structure into phylodynamic (coalescent) approaches to inferring past disease dynamics from genealogies (Rasmussen et al. 2014). Motivated to explain the common discrepancy between phylodynamic inferences and those made from hospital records, the authors apply their new method to a case study of mosquito-borne dengue virus in southern Vietnam. They demonstrate that a spatially structured susceptible-infected-recovered (SIR) model resulted in similar patterns to those seen from the hospitalization data, including large seasonal fluctuations in disease, and that the incorporation of ecological complexity into coalescent models increases the accuracy of inferences to disease demography.

Overall, these recent studies demonstrate the added value of incorporating spatial structure into epidemiological, phylodynamic, and evolutionary/ecological models of infectious disease. Although the current body of theory on the topic offers clear predictions for how spatial structure might influence disease evolution and spread, there remains a paucity of empirical and observational studies testing these key ideas. Moving forward, the more we understand about these eco-evolutionary feedbacks, the better we will be able to manage emerging disease in natural, agricultural, and human populations.
